# Quality Evaluation Indices for Soybean Oil in Relation to Cultivar, Application of N Fertiliser and Seed Inoculation with *Bradyrhizobium japonicum*

**DOI:** 10.3390/foods11050762

**Published:** 2022-03-06

**Authors:** Ewa Szpunar-Krok, Anna Wondołowska-Grabowska

**Affiliations:** 1 Department of Crop Production, University of Rzeszow, Zelwerowicza St 4, 35-601 Rzeszów, Poland; 2 Institute of Agroecology and Plant Production, Wrocław University of Environmental and Life Sciences, Grunwaldzki Sq. 24A, 50-363 Wrocław, Poland; anna.wondolowska-grabowska@upwr.edu.pl

**Keywords:** soybean, cultivar, inoculation, nitrogen fertilization, fatty acids, nutritional indices, human health

## Abstract

Soybean ranks second in production and consumption of vegetable oils worldwide and these are expected to continue to increase. The suitability of soybean oil for specific uses is determined by the fatty acid composition from which a number of indices and indicators can be calculated. The aim of this study was to evaluate the indices of nutritional and health-promoting fat in seeds of soybean cultivars grown in 2016–2019 under the influence of varying doses of N and inoculation with *Bradyrhizobium japonicum*. Omega 3 and Omega 6, unsaturated fatty acids (UFA), saturated fatty acids (SFA), polyunsaturated fatty acids (PUFA), index of desirable fatty acids (DFA), sum of hypercholesterolemic fatty acids (OFA), index of atherogenicity (AI), index of thrombogenicity (TI), oleic desaturation ratio (ODR), linoleic desaturation ratio (LDR), calculated oxidizability value (COX) and the hypocholesterolemic/hypercholesterolemic ratio (HH), saturation fat index (S/P) and ALA/LA, OL/(LA+ALA) ratios and the consumer index (CI) were included. Fat quality indices for soybean seeds were strongly determined by weather conditions. Seeds of the cv. Aldana contained higher amounts of Omega 6 and featured more favourable MUFA/PUFA and OL/(LA+ALA) ratios, while the seeds of the cv. Annushka had more favourable CI and higher ODR, COX and S/P indices. No important differences were observed regarding the effect of nitrogen dose and seed inoculation on the formation of the DFA, OFA, HH, AI, TI and CI indices. The value of the S/P index suggests that higher nitrogen rates (60 kg∙ha^−1^) and the lack of inoculation treatment produce seeds with a more favourable dietary fatty acid balance.

## 1. Introduction

Soybean (*Glycine max* (L.) Merr.) belongs to the crop bean family. Over the period 2017–2021, the global area of soybean increased by 0.74% and it is meant to further increase by another 3.03% by 2027. The amount of soybean consumed increased by about 7.15% during this period and consumption is expected to increase by another 4.95% by 2027 [1]. Soybean is a crucial component of food security around the world since it is a source of protein in human and animal diet, as well as providing cooking oil and biofuels. Meeting the demand for soybean in 2050 in existing crop fields for a total population of 9.7 billion people creates pressure not only to increase crop yield potential but also oil quality [2,3].

Soybean oil is frequently used for food and food processing applications such as confectionery toppings, salad oil, cooking, mayonnaise and food dressing [4,5,6]. Besides, it is used for non-edible applications such as lubricants, biodiesel, cleaners, plastic synthesis, coatings, adhesives, and the production of inks, paints, varnishes and resins [4,7].

The value of soybean seeds depends on their oil content and fatty acid composition [4]. Soybean seeds are a popular source of edible oil, including unsaturated fatty acids such as oleic (OL) and linoleic (LA) acids [8,9,10]. The fatty acids (FAs) contained in soybean oil offer a range of uses [11]. The market demands for soybean oil are opposing. The food industry and the oil and biodiesel industry use the oil with low oxidation capacity, stability of temperature and long shelf life (rich in OL). Whereas oil for direct consumption should feature increased nutraceutical value with a high rate of essential polyunsaturated fatty acids (linoleic acid (LA) and linolenic acid (ALA)) [4]. Due to the fat content, 100 g of soybeans provide 385 kcal. The nutritional value of soybean seeds is enriched by the large amount of unsaturated fatty acids (UFA) [12]. Soybean oil consists of 62% PUFA (54% LA and 23% MUFA (23% OL), 8% ALA), 15% SFA (11% palmitic and 4% stearic) [4]. This oil is a mixture of SFA and unsaturated UFA fatty acids with single (MUFAs) and multiple unsaturated bonds (PUFAs). Since each of the vegetable oils analysed has a specific FA profile, their effects on human health can be evaluated according to the sum of the individual FAs, taking into account their different effects on human health and disease risk [13]. High levels of SFA are associated with diabetes, obesity and hyperlipemia [14]. High intake of total fat has been documented in a global prospective study [15] in Europe ranging 28.5–46.2% ERDI (energy recommended dietary intakes), but also in Africa 13.1–50.7% ERDI and America 25.7–37.2% ERDI. The highest intake of MUFAs 10.9–22.3% ERDI was also reported in Europe [13].

Legumes, including soybean, exhibiting a lower glycemic index compared to other starchy foods [16,17] may help reduce cancer risk [18]. Therefore, diets rich in soy products (soyfoods), considered as potential functional foods, exhibit a number of health-promoting properties [12]. Epidemiological and intervention studies have shown that consumption of legumes is connected to the risk of coronary heart disease [19], type II diabetes [20] as well as obesity [21] and is responsible for lower LDL cholesterol and higher HDL cholesterol [10,22,23]. Soybean oil can lower cholesterol levels in the human body due to its high content of PUFAs [24,25]. A growing interest has been observed in potential protective effects of n3 PUFAs and PUFAs against depressive disorders [26]. In addition to the health-promoting properties of soybean, there are also risks and negative effects that may result from the consumption of soy products. FA accumulation as a result of metabolic disorders and/or improper diet is toxic to many tissues, especially to the liver. Higher concentrations of certain fatty acids, especially PUFAs, can cause cell death. Another observation deals with higher toxicity of SFAs in relation to MUFAs [27].

The health quality of lipids, based on fatty acid composition, is determined by indices [28]. The DFA (neutral and hypocholesterolemic fatty acids) index reports the hypocholesterolemic (total cholesterol-lowering) properties of the lipids analysed [29] as an increase in the hypercholesterolemic index (OFA or HI) may lead to increased cholesterol [30]. The hypocholesterolemic/hypercholesterolemic ratio (HH), can become an indicator of the cholesterol effect of a fat source [30,31]. An increase in the index of atherogenicity (AI) and index of thrombogenicity (TI) provides for lower health-promoting values, since the risk of atherogenicity and thrombogenicity of dietary fat rises with their higher figures. AI and TI are important indicators of the potential impact of fats on cardiovascular health, thrombosis prevention and atherosclerosis [32]. The consumer index (CI) is also referred to as the health quality index [28]. The calculated oxidizability value (COX) and saturation fat index (S/P) indices determine the degree of fat oxidation and saturation. Fat that contains high S/P ratios provides for a more favourable FA balance [30]. The recommended value of the S/P ratio in the human diet is 0.45 [33,34]. The ratio between mono- (OL) and polyunsaturated (LA + ALA) acids serves as a general indicator of oil quality [4,35]. The n-6/n-3 ratio is treated as a key factor for balanced eicosanoid synthesis and its nutritional significance, as well as the dependence of n-6/n-3 ratio values on dietary regime are often discussed. A high intake of vegetable oils rich in n-6 PUFAs and often low intake of marine fish products may result in excessively high values of this ratio, as observed mainly in Western European countries [13].

The FA profile of soybean seeds is significantly connected with genetics [36,37,38] and also depends on the region in which it is grown [8,9,39]. Varietal origin has a clear impact on nutritional traits [35]. Seed yield and soybean oil quality are also affected by agronomic management. In soybean cultivation, seed inoculation with symbiotic bacterial strains is an important agrotechnical procedure. Research to date is mainly devoted to the effect of this treatment on biomass production and nitrogen fixation. The effectiveness of *Bradyrhizobium japonicum* inoculation in aiding nitrogen fixation by soybean plants has been described by many authors [40]. This positive effect of *B. japonicum* inoculation is accompanied by an increase in soybean seed yield [41] by up to 48% and oil by 19% [42].

Seed inoculation can replace the application of nitrogen fertiliser, as demonstrated with the common bean [43]. Furthermore, the effects of N on seed yield, oil content and protein have been well documented [3,44,45], but there are not many studies on the effect of the level of application of nitrogen fertiliser on oil composition, including FA profile.

Inoculation with *B. japonicum* provides FA content of soybean seeds [46]. A similar result of inoculation treatment was obtained by using *Pseudomonas putida* and *Azotobacter chroococcum* [47] After the application of a biofertilizer for seed inoculation in which *B. japonicum*, *Azosprillum lipoferum* and *Pseudomonas putida* were present together, the highest values of oil, oleic acid and linoleic acid contents were obtained, while the application of *Bradyrhizobium* strains increased the values of unsaturated fatty acids in soybean oil [47,48]. Inoculation with *B. japonicum* sv *glycinearum*, increased the content of specific UFAs, for example, the presence of omega-3 essential fatty acid (α-linolenic acid, ALA) and α-oleostearic acid [49].

The combined use of nitrogen fertilisers and seed inoculation can increase legume yields, but it is the FA composition of soybean oil that determines its quality [46,50] and has a significant impact in defining the indices that evaluate its nutritional and health-promoting value.

The aim of this study is the evaluation of the quality and value of soybean oil extracted from seeds depending on cultivar, application of N fertiliser and seed inoculation with *B. japonicum* using indices based on FA profile. This attitude provides the opportunity to compare different vegetable fats and oils, including animal fats and oils, in terms of their healthiness and potential for nutritional use.

## 2. Materials and Methods

### 2.1. Experimental Design

A four-year field experiment (2016–2019) with soybean (*Glycine max* (L.) Merrill) was conducted at the Experiment Station for Cultivar Assessment in Przecław (50°11′ N, 21°29′ E; south-eastern Poland).

It was implemented as a three-factor split-plot design in four replications. (The plot had an area of 19.5 m^2^). Study factors:

- soybean cultivars: Aldana (Plant Breeding Strzelce Sp. z o.o. IHAR group, Strzelce, Poland) and Annushka (Scientific Research Center of Soya Development ”AgeSoya” Sp. z o.o., Huta Krzeszowska, Poland), belonged to the very early maturity group,
- Initrogen fertilizer: 0, 30, 60 kg ∙ ha^−1^ N,
- bacterial inoculant (with symbiotic bacteria *B. japonicum*): control (without bacterial inoculation), HiStick^®^ Soy (BASF, Littlehampton, UK), Nitragina (Institute of Soil Science and Plant Cultivation –State Research Institute, Puławy, Poland).

The cultivation of soybeans was carried out in accordance with the principles of integrated farming. Soybean sowing was performed at the end of April and the beginning of May, at a density of 90 seeds per m^2^, in a field where soybean had not been grown before. The forecrop was spring wheat. Pre-sowing fertiliser with phosphorus and potassium was applied at the rate of 15.3 P and 78.9 K kg ∙ ha^−1^, respectively. The crop was harvested at the end of August and at the beginning of September.

The experiment was carried out on a soil with silt loam (SiL) particle size composition [51], classified as a Fluvic Cambisol (CMfv) [52]. The pH values determined with 1 M KCl indicate a slightly acidic or neutral pH of the soil (pH from 6.38 to 6.82). The content of available phosphorus (P from 101 to 214 mg · kg^−1^ DM of soil) was very high, potassium (K from 128 to 273 mg · kg^−1^ DM of soil)—very high or medium, magnesium (Mg from 134 to 243 mg · kg^−1^ DM of soil)—very high or high, manganese (Mn from 118 to 402 mg · kg^−1^ DM of soil) and zinc (Zn from 10.7 to 13.8 mg · kg^−1^ DM of soil)—medium and copper (Fe from 3.82 to 11.6 mg · kg^−1^ DM of soil)—high or medium.

The detailed methodology of the field experiment is presented in the work of Szpunar-Krok et al. [53].

### 2.2. Weather Conditions

Meteorological data are given according to the records of the Experimental Station for Cultivar Assessment in Przecław. Weather conditions in the course of the soybean growing season (April–September) varied depending on the year of the study, and also in individual months (Table 1). Based on Sielianinov’s hydrothermal index (K), the 2016 growing season was described as optimal, 2017 was humid, 2018 was relatively dry and 2019 was relatively humid. Extremely dry weather occurred in April 2018 and June 2019, while July 2016 and May and September 2017 were very humid and April 2017 and May 2019 were very humid.

**Table 1:** The hydrothermal index (K) during the growing season of soybean.

Years	Months						Mean for Apr–Sep
	Apr	May	Jun	Jul	Aug	Sep	
2016	1.86 (rh) *	0.96 (d)	0.43 (vd)	2.59 (vh)	1.25 (rd)	0.89 (d)	1.33 (o)
2017	3.79 (eh)	2.88 (vh)	0.80 (d)	0.80 (d)	1.50 (o)	2.94 (vh)	2.12 (h)
2018	0.42 (vd	1.43 (o)	0.94 (d)	1.88 (rh)	1.70 (rh)	0.88 (d)	1.21 (rd)
2019	2.93 (vh)	4.63 (eh)	0.31 (ed)	0.82 (d)	1.47 (o)	1.86 (rh)	2.00 (rh)

* Ranges of Sielianinov index (K) values proposed by Skowera et al. [54]: K ≤ 0.4 extremely dry (ed), 0.4 < K ≤ 0.7 very dry (vd), 0.7 < K ≤ 1.0 dry (d), 1.0 < K ≤ 1.3 relatively dry (rd), 1.3 < K ≤ 1.6 optimal (o), 1.6 < K ≤ 2.0 relatively humid (rh), 2.0 < K ≤ 2.5 humid (h), 2.5 < K ≤ 3.0 very humid (vh) and K > 3.0 extremely humid (eh).

### 2.3. Analytical Methods

The FA profile of soybean seeds was determined by gas chromatography with FID flame ionization detection (Clarus 580, Perkin-Elmer, Shelton, WA, USA) using a ZB-WAX column (30 m × 0.25 mm id, 0.25 μm film thickness). Qualitative interpretation of the chromatograms was performed by comparing the retention times of fatty acid methyl esters in the test sample with those of Supelco 37 fatty acid methyl ester matrices. The detailed methodology for determining the FA profile and its content in soybean seeds is given in the work by Szpunar-Krok et al. [53].

The indices presented in Table 2 were used to assess the nutritional value of FAs contained in soybean seeds and to investigate the possibility of their use in the prevention and treatment of diseases.

**Table 2:** The indicators of nutritional quality of soybean oil.

Indices	Calculation Formula	Application
Omega 3	(C18:3n3 + C18:4n3 + C20:4n3 + C20:5n3 + C22:5n3 + C24:5n3 + C24:6n3 + C22:6n3)	
Omega 6	(C18:2n6 + C18:3n6+ C20:2, C20:3n6 +C20:4n6)	
Omega 6/Omega 3	ΣOmega 6/ΣOmega 3	[55,56,57,58]
MUFA/PUFA	ΣMUFA/ΣPUFA	[59]
UFA/SFA	ΣUFA/ΣSFA	[32]
PUFA/SFA	ΣPUFA/ΣSFA	[32,60]
ALA/LA— α-Linolenic acid/Linoleic acid ratio	C18:3 n-3/C18:2 n-6	[61]
DFA—Index of desirable fatty acids	C18:0+∑UFA	[29,62]
OFA—Sum of hypercholesterolemic fatty acids	C14:0+C16:0	[63]
HH—Hypocholesterolemic/Hypercholesterolemic ratio	(C18:1n-9 + C18:2n-6 + C20:4n-6 + C18:3n- 3 + C20:5n-3 + C22:5n-3 + C22:6n-3)/(C14:0 + C16:0)	[31,32,60,64,65,66,67]
AI—Index of atherogenicity	$\frac{C12:0+4\;\left(C14:0\right)+\;C16:0}{\sum \mathrm{MUFA}\;+\sum \left(n–6\right)+\sum \left(n–3\right)}$	[32,64,65,68,69,70,71]
TI—Index of thrombogenicity	$\frac{C14:0+\;C16:0+\;C18:0}{0.5\sum \mathrm{MUFA}\;+0.5\sum \left(n–6\;\mathrm{PUFA}\right)+3\sum \left(n–3\;\mathrm{PUFA}\right)+\frac{\sum \;\left(n–3\right)}{\sum \;\left(n–6\right)}}$	[64,65,68,69,70,72]
CI—Consumer index	(C18:3+ C20:5+ C22:6)	[73]
ODR—Oleic desaturation ratio	$\left[\%\;C18:\;2+\left(\frac{\;\%\;C18:\;3}{\%\;C18:\;1}\;\right)+\;\%\;C18:\;2+\;\%\;C18:\;3\right]\times 100$	[74,75]
LDR—Linoleic desaturation ratio	$\left[\frac{\%\;C18:\;3}{\%\;C18:\;2\;}+\;\%\;C18:\;3\right]\times 100$	[74,75]
COX—Calculated oxidizability value	$\frac{\left[1\;\left(18:\;1,\;\%\right)+10.3\;\left(18:\;2,\;\%\right)+21.6\;\left(18:\;3,\;\%\right)\right]}{100}$	[75,76,77,78]
S/P—Saturation fat index	(C14:0+C16:0+C18:0)/(MUFA+PUFA)	[68]
OL/(LA+ALA)	18: 1/(18:2+ C18:3)	[4]

### 2.4. Statistical Analyses

The results of the study were statistically processed using analysis of variance (three-way ANOVA). In order to determine and verify the relationship, Tukey’s post-hoc range test was performed at *p* ≤ 0.05. The TIBCO Statistica 13.3.0 software (TIBCO Software Inc., Palo Alto, CA, USA) was used for the calculations.

## 3. Results and Discussion

### 3.1. Omega 3, Omega 6, Omega 6/Omega 3

Increased intake of (Omega 3) PUFAs is associated with reduced risk of cardiovascular morbidity and mortality [79,80,81]. According to FAO/WHO, the recommended optimal daily intake of Omega 3 and Omega 6 should maintain the ratio, such as 5-10:1 [82]. A lower ratio of Omega 6/Omega 3 FAs is more desirable for reducing the risk of many diseases [57].

The chemical composition of seeds, including the FA profile, is primarily genetically determined [37,83,84]. The effect of cultivar on the fatty acid profile is confirmed by the conducted experiment. The study showed a considerable impact on soybean cultivar regarding the content of Omega 3 and Omega 6 acids in seeds (Table 3). Seeds of the cv. Annushka had a noticeably higher content of Omega 3 acids, which may have a beneficial effect on human health, and a higher level of Omega 6, which is not desirable in human nutrition. These values were higher, respectively, by 0.25 and 1.0 g∙100 g seeds^−1^ in comparison with the cv. Aldana, but the ratio of Omega 6 to Omega 3 acids in the seeds of these cultivars did not differ significantly.

Bacterial inoculation can completely replace chemical fertiliser in various legumes, as it increases nitrogen fixation in the soil and nitrogen uptake by the plants and thus affects the increase in yield and nitrogen content in the plant [85]. Silva et al. [86] showed that inoculation of *B. japonicum* sv *glycinearum* caused an increase in total fatty acids and this was due to an increase in MUFAs and PUFAs. In the conducted study, application of nitrogen fertiliser to soybean as well as pre-sowing inoculation of seeds with symbiotic bacteria *B. japonicum* did not influence the content of Omega 3 and Omega 6 acids in the seeds, nor the formation of the Omega 6/Omega 3 ratio.

FA composition at maturity results from metabolic pathways and is strongly regulated by management and environmental conditions during seed filling [35,87,88]. Environmental conditions during seed development affect component accumulation and can also cause a decrease in oil content [4]. In the conducted experiment, the weather conditions were a factor that strongly determined the fat quality indicators in soybeans. In the very warm and relatively humid 2019, seeds with the highest content of Omega 3 acids were harvested. Seeds from the 2019 harvest had the highest content of Omega 3 acids, significantly higher than the seeds in 2017 and 2018 which had the lowest content, by 13.5 and 15.5%, respectively. The warm year of 2016, with optimal moisture conditions, favoured the unfavourable accumulation of Omega 6 acids. Indeed, soybean seeds in 2016 contained the most Omega 6 acids, 5.8% more in relation to seeds harvested in 2018, which contained the lowest values. In 2017 and 2018, soybean seeds had a value of Omega 6/Omega 3 ratios that were significantly the highest, which is an unfavourable phenomenon in terms of the assessment of the quality of food raw materials.

Statistical analysis points at a significant interaction between the cultivar and the year of the experiment in determining the content of the Omega 3 acids and Omega 6/Omega 3 acid ratio (Table 4). Seeds of the cv. Annushka had a significantly higher content of Omega 3 acids in 2019 compared to seeds of the same cultivar in 2018 and seeds of the cv. Aldana from 2017 and 2018 harvest by 17.9, 21.1 and 18.6%, respectively, and lower value of Omega 6/Omega 3 ratio (by 15.7%) in relation to the cv. Aldana in 2017.

However, statistical analysis of the results of the four-year study did not find a significant interaction effect of nitrogen fertiliser application and year of study (Table S1), seed inoculation with symbiotic bacteria *B. japonicum* and year of study (Table S2), cultivar and nitrogen fertiliser application (Table S3), cultivar and inoculation (Table S4), as well as nitrogen fertilization and inoculation (Table S5) on the formation of the content of Omega 3 and Omega 6 acids and the Omega 6/Omega 3 ratio in the seeds. The results of the relationship between Omega 3 and Omega 6 acids in the study carried out were within the range of values recommended by FAO/WHO [82].

### 3.2. MUFA, PUFA, UFA, SFA

Omega-3 PUFAs include a-linolenic acid (ALA; 18:3 Omega 3), stearidonic acid (SDA; 18:4 Omega 3), eicosapentaenoic acid (EPA; 20:5 Omega 3), docosapentaenoic acid (DPA; 22:5 Omega 3) and docosahexaenoic acid (DHA; 22:6 Omega 3). They are thought to have effects on cardiovascular disease, diabetes, cancer, Alzheimer’s disease, dementia, depression, visual and neurological development, and maternal and child health. Although many health benefits of Omega 3 PUFAs have been described in the literature, there is also some controversy regarding their efficacy and some benefits to human health [89]. Changes involving the amount of SFAs and increasing the sum of n-3 PUFAs and decreasing the ratio of n-6/n-3 PUFAs should be considered nutritionally important [73]. Low saturated fat intake and an increased PUFA/SFA ratio are associated with lower risk of coronary heart disease in humans. Therefore, the PUFA/SFA ratio is one of the main parameters used to assess the nutritional quality of the lipid fraction of foods. Guidelines recommend a PUFA/SFA ratio above 0.45 [90]. Large differences in the UFA profile are more determined by environmental than genotypic conditions [88,91].

The experiment showed a considerable effect of soybean cultivar on the formation of MUFA/PUFA ratios, but no significant effect of cultivar on UFA/SFA, PUFA/SFA and C18:3n3/C18:2n6 ratios (Table 3). Seeds of the cv. Aldana were characterized by a significantly lower value of MUFA/PUFA ratio by 12.8%, which is a favourable indicator with respect to fat quality and its positive effects on human health.

Inoculation with *B. japonicum* resulted in a significant increase in PUFA content compared to the control. Inoculation with *B. japonicum* resulted in a significant increase in MUFA content [86]. In the conducted study, application of nitrogen fertiliser to soybean as well as pre-sowing seed inoculation with the symbiotic bacteria *B. japonicum* did not affect the formation of MUFA/PUFA, UFA/SFA and PUFA/SFA acid ratios in the seeds of the test cultivars.

The seeds harvested in 2018 were distinguished by the highest values of MUFA/PUFA, UFA/SFA and PUFA/SFA ratios, which, compared to the other combinations, is a very favourable phenomenon as far as the evaluation of seed quality and its impact on human health are concerned.

Large differences in the UFA profile were modified by environmental conditions [88]. The sensitivity of UFA composition to changes in thermal conditions is well documented, that is, typically, an inverse relationship between polyunsaturated fatty acids and temperature during soybean seed development. UFA profile responds differently to temperature and field water availability during seed filling with both of these climatic factors showing positive effects on UFA [4].

Statistical analysis proves a significant interaction between cultivar and year of experiment in determining the MUFA/PUFA ratio (Table 4). Seeds of the cv. Aldana in 2018 had the highest value of the MUFA/PUFA ratio, higher by 40% compared to the lowest value of this ratio found in seeds of the cv. Annushka in 2016.

In the four-year experiments, there was no significant interaction effect of nitrogen fertiliser application and test year (Table S1), seed inoculation with symbiotic bacteria *B. japonicum* and test years (Table S2), nitrogen and cultivar fertiliser application (Table S3), inoculation and cultivar (Table S4) or nitrogen fertiliser application and inoculation (Table S5) on the formation of the MUFA/PUFA, UFA/SFA and PUFA/SFA ratios.

### 3.3. ALA/LA

FA composition of soybean oil is dominated by polyunsaturated L acid. Due to the presence of two isolated double bonds, it is sensitive to oxidation and degradation during heat treatment [92,93]. The LA acid content of soybean oil is slightly lower than that of corn and sunflower oils, but more than twice that of canola oil. Soybean has a relatively high content of ALA acid [12]. LA (C18:2, Omega 6, LA) and ALA acid (C18:3, Omega 3, ALA) are considered essential and particularly beneficial to health. These acids represent 54% and 7,5 % of the soybean FA pool, respectively [12]. Muhammad Azam et al. [94] showed that the levels of LA and OL acid differ to a highly significant degree between cultivars. This opinion is not supported by our own study as the compared cultivars showed similar qualitative characteristics. Soybean seed inoculation treatment with the *B. japonicum* strain results in better nitrogen nutrition and improves product quality due to increased nitrogen content in seeds and increased UFA content in soybean oil [95].

Studies by other authors indicate that both inoculation and N fertiliser application increase the level of UFAs (LA and OL) [96]. In one study, nitrogen fertiliser application to soybean, as well as pre-sowing seed inoculation with symbiotic bacteria *B. japonicum*, did not affect the ALA/LA acid ratios [39] and showed that the content of LA and ALA acids is significantly modified by the course of weather variation during the years of the study. In the conducted experiment, statistical analysis allows the observation of a significant interaction between year and cultivar in determining the ALA/LA ratio (Table 4). In 2017 and 2018, the soybean seeds had lowest ALA/LA, which was a significant difference. In both cultivars tested, the highest ALA/LA ratio value was found in the 2019 seeds.

The most desirable ratio of ALA to LA for human health should be in balance [57]. In our study, the most favourable ratios of these acids were recorded in years with different climatic conditions and with no logical correlations—2017 (very cool, humid) and 2018 (very warm, relatively dry).

### 3.4. DFA, OFA, HH

The DFA value gives information about hypocholesterolemic properties (lowering the level of total cholesterol) of the lipids analysed [29]. The experiment showed a significant effect of weather in the years of the study and no significant effect of nitrogen application rates and seed inoculations with *B. japonicum* on the formation of the DFA, OFA and HH indices indicating the quality of fat in soybean seeds (Table 5). In 2016 and 2018, the value of the DFA index value was significantly higher than in 2017 by 1.6 and 3.0%, respectively. In 2016 and 2017, the OFA index was significantly higher than in 2018 by 12.3%, while the HH index values in 2016 and 2017 were significantly lower than in 2018 by 14.8 and 16.4%, respectively. It is believed that the HH index is one of the best indicators to determine the nutritional quality of the product consumed [32].

The index of desirable acids DFA was most favourable in 2016 and 2018, which were characterized as very warm or warm with the optimal or relatively dry moisture conditions. The OFA index value, as a sum of hypercholesterolemic saturated fatty acids, was the least favourable for seeds obtained in the warm season with an optimal course of precipitation in 2016 and in the very cool and humid 2017. The most favourable HH index featured soybean seeds harvested in the very warm and relatively dry 2018, so they can be considered as seeds with higher health-promoting value compared to seeds obtained in the other years of the study.

The experiment did not show any significant effect of the interaction of cultivar and year of testing on the formation of DFA, OFA and HH (Table 6).

### 3.5. AI, TI, CI

Of the SFAs, only those with chain lengths of 12, 14 or 16 carbon atoms have cholesterol-raising effects and, therefore, are atherogenic (defining the AI index). SFAs with chain lengths of 14, 16 or 18 carbon atoms are considered thrombogenic (defining the TI index) [32]. The increases in AI and TI result in lower health-promoting values, whereas lower values of both indices speak for better nutritional quality of fatty acids; therefore, diets with low AI and TI values may reduce the potential risk of coronary heart disease (CHD) [32,60]. AI and TI are believed to be the best indicators of the nutritional quality of foods (especially fish) [32]. Among the indices that have been calculated to determine the lipid quality in *G. max* seeds, the varietal factor only showed significant variation in the CI index (Table 5). Seeds of the cv. Annushka had a considerably higher value of this index of fat quality with an increase of 2.6% compared to the cv. Aldana.

The experiment showed a significant effect of weather during the study years and no significant effect of nitrogen application rates and *B. japonicum* seed inoculation on the values of AI, TI and CI indices, which indicate the quality of fat in soybean seeds.

In 2016 and 2017, the TI index reached a significantly higher value compared to 2018 by 9.0 and 16.5%, respectively, suggesting the acquisition of soybean seeds with reduced health-promoting value, while the oil contained in seeds from the 2018 harvest had a significantly lower AI index value by 14.9%, thus indicating better nutritional quality of the FAs contained in soybean seeds. On the other hand, the CI index value describing the oil quality of soybean seeds harvested in 2019 was significantly higher than in 2017 and 2018 by 12.8 and 15.4%, respectively. Assuming that the CI value should represent up to 3% of the total fatty acid pool [28], it should be noted that in the conducted experiment, it exceeded this index by about three times, making the soybean seeds not the best raw consumer material.

There was no significant interaction effect of nitrogen fertiliser application and test year (Table S6), seed inoculation with symbiotic bacteria *B. japonicum* and test years (Table S7), cultivar and nitrogen fertiliser applied (Table S8), cultivar and seed inoculation (Table S9), nitrogen fertiliser application and inoculation (Table S10) on the formation of the DFA, OFA, HH, AI, TI and CI indices.

### 3.6. ODR, LDR, COX, S/P, OL/(LA + ALA)

The ODR, LDR and COX ratios provide information on the degree of destructuration of oleic acid (ODR), linoleic acid (LDR) and the degree of oxidation of OL, LA and ALA acids in oxidized ester mixtures (COX). The value of the P/S ratio indicates whether it is appropriate for the human diet. Fat with high S/P ratios indicates a “healthier” balance of FAs [33,34].

Statistical analysis proved a significant effect of soybean cultivar on the value of seed fat quality indices ODR, COX, S/P and OL/(LA + ALA) (Table 7). Significantly higher ODR, COX and S/P index values were obtained in the seeds of the cv. Annushka compared to the cv. Aldana (by 2.8, 1.6 and 5.0%, respectively), while the value of the OL/(LA+ALA) ratio was lower by 11.3%.

Nitrogen fertiliser application and seed inoculation with *B. japonicum* bacteria affected only the fat quality parameter S/P. It was found that the increase in the pre-sowing dose of nitrogen caused an increase in the value of the S/P index, while in the variant with HiStick^®^Soy inoculation this parameter reached a significantly lower value compared to the control (without inoculation), which may suggest that the application of fertiliser with higher doses of nitrogen and lack of seed inoculation allows seeds with a more favourable balance of FAs in the diet to be obtained.

In the experiment conducted here, the factor most strongly determining all fat quality indices in Table 7 was the weather pattern. In 2016 and 2019, soybean seeds had the highest values of ODR and COX indices compared to 2017 and 2018, and the lowest OL/(LA+ALA) ratio. The 2019 seed also had the highest value of the LDR index.

The recommended S/P ratio should be close to the value of 0.45, which is considered appropriate for the human diet [33,34]. Therefore, the seeds obtained from each combination not exceeding this ratio should be considered to have a good balance of fatty acids. Seeds of the cv. Annushka showed a more favourable ratio.

Statistical analysis appoints to a significant interaction of years of experiment and cultivar in determining the ODR, LDR S/P indices and OL/(LA+ALA) ratio (Table 8). The highest value of the ODR index was obtained for the cv. Annushka in 2016. The LDR fat quality index values were the highest in both the 2019 cultivars. On the other hand were the S/P index values in 2017 and 2016 for the cv. Annushka and in 2019 for the cv. Aldana.

**Figure 1:**
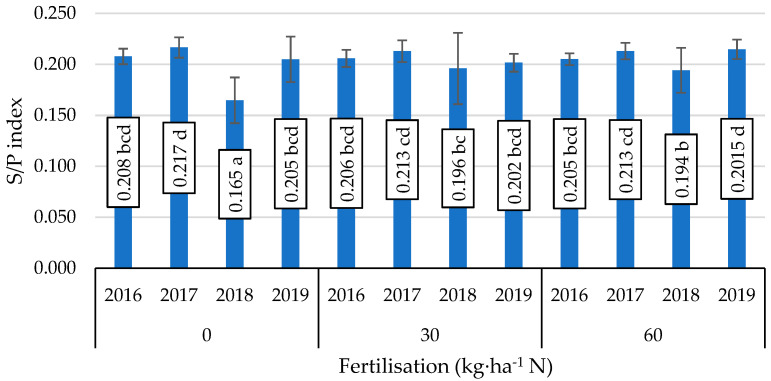
Value of the saturation fat index (S/P), mean values for interaction nitrogen fertiliser application × years. Mean values ± SD. Means followed by different superscripts show significant differences (*p* < 0.05) according to the post-hoc Tukey test. Differences significant at: *p*  <  0.001.

A considerable interaction of fertiliser application and year of study was only shown in the formation of the S/P index (Figure 1). The lowest value of this fat quality parameter was obtained in 2018 in soybean seeds not given an application of nitrogen fertiliser, while the highest values of this index were also reported in the absence of nitrogen fertiliser in 2017 and under the influence of nitrogen fertiliser in 2019.

**Figure 2:**
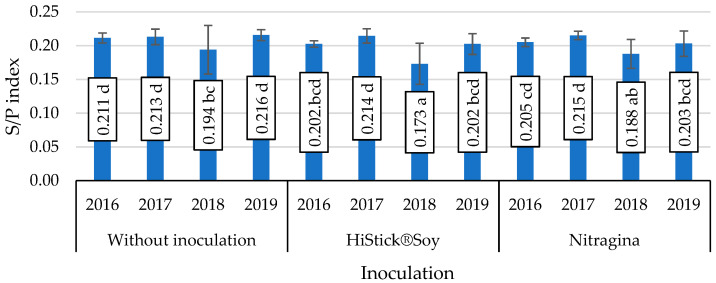
Value of the saturation fat index (S/P), mean values for interaction inoculation × years. Mean values ± SD. Means followed by different letters show significant differences (*p* < 0.05) according to the post-hoc Tukey test. Differences significant at: *p*  <  0.05.

The interaction of seed inoculation with *B. japonicum* bacteria and year of study was also significant in determining the S/P index (Figure 2). The lowest values of this index in soybean seeds, which were significant, were obtained in 2018 on subjects inoculated with HiStick^®^Soy and Nitragina bacterial preparations.

A significant interaction of cultivar and seed inoculation with *B. japonicum* bacteria was shown in the formation of the COX and S/P indices (Table 9). In the cv. Annushka after application of the Nitragina bacterial preparation and in the variant without inoculation, the COX index reached significantly higher values compared to the cv. Aldana inoculated with Nitragina, by 3.2 and 2.4%, respectively. In case of the S/P index, the highest value was obtained in the cv. Annushka in the control variant without inoculation, which was 12.9% higher than its lowest value found in seeds of the cv. Aldana treated with the HiStick^®^ Soy preparation.

**Figure 3:**
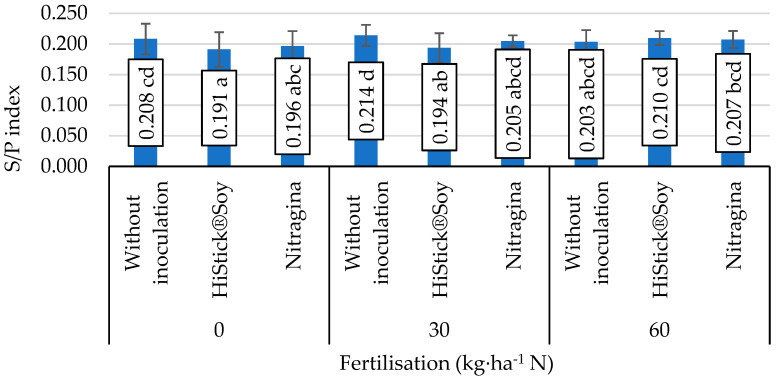
Value of the saturation fat index (S/P), mean values for interaction nitrogen fertiliser application × inoculation. Mean values ± SD. Means followed by different superscripts show significant differences (*p* < 0.05) according to the post-hoc Tukey test. Differences significant at: *p*  <  0.01.

The lack of application of nitrogen fertiliser and pre-sowing use of nitrogen fertiliser in the dose of 30 kg∙ha^−1^ N combined with seed inoculation with HiStick^®^Soy and Nitragina preparations caused a decrease in the S/P index values compared to objects where inoculation was not applied (Figure 3). Increasing the nitrogen dose to 60 kg∙ha^−1^ N and the inoculation of seeds with *B. japonicum* bacteria had no effect on the development of this index.

In the case of the ODR, LDR and COX indices in the experiment, there was no significant interaction between the cultivar and application of nitrogen fertiliser (Table S11), application of nitrogen fertiliser and year of study (Table S12), years of experiment and inoculation (Table S13), application of nitrogen fertiliser and inoculation (Table S14).

### 3.7. OL/(LA + ALA)

The OL/(LA + ALA) ratio is a well-known indicator of oil quality. LA acid is a very important PUFA that must be present in the diet because it is not synthesised by the human body [97]. Higher Ol acid content and lower LA and ALA acid content are beneficial due to fat stabilisation [98]. The ratio of monounsaturated to polyunsaturated OL/(LA+ALA) was different between cultivars with a maximum value 18 % higher than the minimum, but without the effect of N fertiliser application [37].

Statistical analysis proves a significant interaction of cultivar and years of experiment in shaping the OL/(LA+ALA) ratio (Table 8). The highest value of the FA OL/(LA+ALA) ratio was recorded in 2018 in the cv. Aldana.

The level of accumulation of OL, LA and ALA acids and the ratio OL/(LA+ALA) results varied in response to temperature and water availability in the field during seed filling. Drought and water deficit caused a linear increase in OL and the OL/(LA+ALA) ratio and a linear decrease in LA and ALA. Studies have shown that OL concentration increases and LA and ALA decreases with increasing temperature during seed filling. Under moderate and cool conditions, the level of essential fatty acids (LA, ALA) increases and, under warm conditions, the OL/(LA+ALA) ratio increases (OL increases and ALA decreases with increasing temperature) [4].

Water deficit during seed filling increases OL concentration, while LA and ALA concentration decreases [99]. OL acid content and OL/(ALA + LA) ratio increase while LA acid content decreases with increasing temperature. The oil quality index OL/(LA+ALA) shows greater modification due to environmental conditions than due to genotypic conditions [88]. In our experiment, this index was modified by both climatic and varietal factors. Seeds with the highest fat stabilization were obtained in a very warm and relatively dry year (2018). For the OL/(LA+ALA) ratio in the years of study, there was no significant interaction of nitrogen fertilizer and cultivar (Table S11), nitrogen fertiliser application and year of study (Table S12), inoculation and test years (Table S13) or nitrogen fertiliser application and inoculation (Table S14).

## 4. Conclusions

The suitability of soybean oil for a specific application is determined by its fatty acid composition, which affects its physical and chemical properties and human health. The profile of fatty acids depends on the cultivar and this determines the values of indices determining fat quality. Seeds of the cv. Aldana had a more favourable Omega 6 content and MUFA/PUFA and OL/(LA+ALA) ratios, while seeds of the cv. Annushka had a more favourable consumer index (CI) and significantly higher values of the ODR, COX and S/P indices.

The level of N fertiliser application and pre-sowing inoculation of seeds of *Bradyrhizobium japonicum* have no effect on the indices. The values of the S/P index are the only ones which suggest that higher rates of nitrogen (60 kg · ha^−1^) and no inoculation treatment produces seeds with a more favourable balance of fatty acids in the diet. In our opinion, this warrants further research in this area.

Climatic conditions were the determinants of all the indicators of soybean oil quality assessment. The course of plant vegetation growth in very warm and relatively humid conditions (2019) allowed seeds with the highest content of Omega 3 acids and Omega 6/Omega 3 ratio to be obtained. Soybean vegetation growth falling in a very warm and relatively dry period (2018) resulted in seeds with values of Omega 6/Omega 3 ratios that were significantly the highest, which is an unfavourable phenomenon in terms of assessing the quality of the food raw material. Seeds harvested in the same growing season were distinguished by the highest values of MUFA/PUFA, UFA/SFA, PUFA/SFA ratios and the lowest sum of Omega 6 acids, which is a very favourable phenomenon with respect to the evaluation of seed quality and its impact on human health. The most desirable ALA to LA ratio was recorded during a very cool and humid growing season for the plants. Very warm or warm years with optimal or relatively dry moisture conditions allowed the most favourable index of desirable DFA acids to be achieved. Soybean seeds harvested in a very warm and relatively dry year had the most favourable ratio of the HH index. Therefore, they can be considered as seeds of higher health-promoting value compared to seeds obtained in cool and wet years.

The results of the research can be used in the process of commodity production management aimed at obtaining raw material of the desired quality, which is important in terms of nutrition and promotion of good health.

## Figures and Tables

**Table 3:** The content of Omega 3 and Omega 6 (g FA 100 g seeds^−1^) as well as the ratio of Omega 6/Omega 3, MUFA/PUFA, PUFA/SFA, UFA/SFA and ALA/LA in *G. max* seeds; mean values for factors.

Factors	Omega 3 (g∙100 g seeds^−1^)	Omega 6 (g∙100 g seeds^−1^)	Omega 6/Omega 3	MUFA/PUFA	UFA/SFA	PUFA/SFA	ALA/LA
Cultivars							
Aldana	9.00 ^a^ ± 0.69	52.5 ^a^ ± 1.62	5.86 ± 0.38	0.334 ^b^ ± 0.056	4.62 ± 0.61	3.46 ± 0.38	0.169 ± 0.012
Annushka	9.25 ^b^ ± 0.74	53.5 ^b^ ± 1.48	5.81 ± 0.40	0.296 ^a^ ± 0.041	4.40 ± 0.48	3.39 ± 0.35	0.170 ± 0.012
Fertilisation (kg∙ha^−1^ N)							
0	9.08 ± 0.82	52.9 ± 1.70	5.87 ± 0.42	0.320 ± 0.061	4.56 ± 0.65	3.45 ± 0.41	0.169 ± 0.013
30	9.13 ± 0.65	52.9 ± 1.60	5.81 ± 0.35	0.314 ± 0.049	4.44 ± 0.57	3.38 ± 0.37	0.170 ± 0.012
60	9.17 ± 0.71	53.2 ± 1.62	5.82 ± 0.41	0.312 ± 0.047	4.53 ± 0.46	3.45 ± 0.31	0.170 ± 0.013
Inoculation							
Without inoculation	9.12 ± 0.79	52.9 ± 1.63	5.83 ± 0.40	0.317 ± 0.061	4.47 ± 0.55	3.39 ± 0.33	0.170 ± 0.013
HiStick^®^Soy	9.09 ± 0.72	53.0 ± 1.76	5.85 ± 0.38	0.318 ± 0.052	4.52 ± 0.54	3.43 ± 0.36	0.169 ± 0.012
Nitragina	9.17 ± 0.68	53.1 ± 1.53	5.82 ± 0.41	0.312 ± 0.043	4.53 ± 0.61	3.45 ± 0.41	0.170 ± 0.013
Years							
2016	9.50 ^a^ ± 0.28	54.8 ^c^ ± 0.72	5.77 ^b^ ± 0.13	0.272 ^a^ ± 0.022	4.51 ^a^ ± 0.17	3.54 ^b^ ± 0.11	0.171 ^b^ ± 0.003
2017	8.62 ^a^ ± 0.30	52.4 ^ab^ ± 0.50	6.09 ^c^ ± 0.25	0.315 ^b^ ± 0.013	4.08 ^a^ ± 0.20	3.10 ^a^ ± 0.13	0.160 ^a^ ± 0.005
2018	8.42 ^b^ ± 0.47	51.6 ^a^ ± 1.86	6.15 ^c^ ± 0.31	0.386 ^c^ ± 0.049	5.07 ^b^ ± 0.77	3.65 ^b^ ± 0.52	0.160 ^a^ ± 0.009
2019	9.97 ^c^ ± 0.29	53.1 ^b^ ± 1.02	5.33 ^a^ ± 0.13	0.289 ^a^ ± 0.018	4.39 ^a^ ± 0.34	3.40 ^ab^ ± 0.27	0.186 ^c^ ± 0.005
Mean	9.13 ± 0.72	53.0 ± 1.62	5.83 ± 0.39	0.315 ± 0.052	4.51 ± 0.56	3.54 ± 0.11	0.169 ± 0.012
Significances							
Cultivar	*	**	NS	***	NS	NS	NS
Fertilisation	NS	NS	NS	NS	NS	NS	NS
Inoculation	NS	NS	NS	NS	NS	NS	NS
Years	***	***	***	***	***	**	***

* Mean values ± SD. MUFA—monounsaturated fatty acids; PUFA– polyunsaturated fatty acids; UFA– unsaturated fatty acids; SFA– saturated fatty acids; ALA/LA—C18:3 n-3/C18:2 n-6 acid ratio. Means in a column followed by different superscripts show significant differences (*p* < 0.05) according to the post-hoc Tukey test. Differences significant at: *** *p*  <  0.001; ** *p*  <  0.01; * *p*  <  0.05; NS not significant.

**Table 4:** The content of Omega 3 and Omega 6 (g FA 100 g seeds^−1^) as well as the ratio of Omega 6/Omega 3, MUFA/PUFA, PUFA/SFA, UFA/SFA and ALA/LA in *G. max* seeds; mean values for interaction cultivar × years.

Cultivar	Year	Omega 3 (g∙100 g seeds^−1^)	Omega 6 (g∙100 g seeds^−1^)	Omega 6/ Omega 3	MUFA/PUFA	UFA/SFA	PUFA/SFA	ALA/LA
Aldana	2016	9.30 ^bc^ ± 0.20	54.2 ± 0.34	5.83 ^b^ ± 0.13	0.293 ^bc^ ± 0.007	4.60 ± 0.13	3.56 ± 0.10	0.170 ^bc^ ±0.003
	2017	8.36 ^a^ ± 0.11	52.6 ± 0.53	6.30 ^c^ ± 0.12	0.324 ^cd^ ± 0.009	4.21 ± 0.17	3.18 ± 0.12	0.155 ^a^ ± 0.002
	2018	8.53 ^a^ ± 0.60	50.6 ± 1.67	5.94 ^b^ ± 0.28	0.420 ^e^ ± 0.039	5.33 ± 0.83	3.76 ± 0.59	0.166 ^b^ ± 0.008
	2019	9.82 ^cd^ ± 0.30	52.6 ± 0.94	5.36 ^a^ ± 0.17	0.301 ^bc^ ± 0.017	4.33 ± 0.25	3.33 ± 0.20	0.186 ^d^ ± 0.006
Annushka	2016	9.69 ^cd^ ± 0.18	55.4 ± 0.39	5.71 ^b^ ± 0.11	0.252 ^a^ ± 0.005	4.41 ± 0.16	3.53 ± 0.12	0.173 ^c^ ± 0.002
	2017	8.87 ^ab^ ± 0.17	52.2 ± 0.40	5.88 ^b^ ± 0.14	0.306 ^bc^ ± 0.009	3.95 ± 0.14	3.03 ± 0.09	0.165 ^b^ ± 0.002
	2018	8.31 ^a^ ± 0.31	52.7 ± 1.38	6.35 ^c^ ± 0.18	0.352 ^d^ ±0.030	4.80 ± 0.64	3.55 ± 0.45	0.154 ^a^ ± 0.004
	2019	10.12 ^d^ ± 0.21	53.7 ± 0.83	5.31 ^a^ ± 0.08	0.277 ^ab^ ±0.006	4.44 ± 0.41	3.48 ± 0.31	0.187 ^d^ ±0.003
Significances								
Cultivar x Years		*	NS	***	*	NS	NS	*

* Mean values ± SD. MUFA—monounsaturated fatty acids; PUFA—polyunsaturated fatty acids; UFA– unsaturated fatty acids; SFA– saturated fatty acids; ALA/LA—C18:3 n-3/C18:2 n-6 acid ratio. Means in a column followed by different letters show significant differences (*p* < 0.05) according to the post-hoc Tukey test. Differences significant at: *** *p*  <  0.001; * *p*  <  0.05; NS not significant.

**Table 5:** The DFA, OFA, HH, AI, TI and CI indices of lipid quality in *G. max* seed; mean values for factors.

Factors	DFA	OFA	HH	AI	TI	CI
Cultivars						
Aldana	85.2 ± 1.69	13.0 ± 1.37	6.63 ± 1.01	0.166 ± 0.021	0.434 ± 0.047	9.07 ^a^ ± 0.67
Annushka	84.6 ± 1.58	13.5 ± 1.37	6.36 ± 0.84	0.173 ± 0.022	0.452 ± 0.046	9.31 ^b^ ± 0.75
Fertilisation (kg∙ha^−1^ N)						
0	85.1 ± 1.87	13.1 ± 1.59	6.63 ± 1.17	0.167 ± 0.024	0.440 ± 0.054	9.15 ± 0.81
30	84.6 ± 1.70	13.5 ± 1.53	6.37 ± 0.95	0.173 ± 0.024	0.450 ± 0.052	9.19 ± 0.65
60	85.0 ± 1.37	13.2 ± 0.97	6.48 ± 0.62	0.168 ± 0.016	0.440 ± 0.034	9.23 ± 0.71
Inoculation						
Without inoculation	84.8 ± 1.63	13.5 ± 1.38	6.35 ± 0.85	0.173 ± 0.022	0.450 ± 0.050	9.18 ± 0.79
HiStick^®^Soy	85.0 ± 1.58	13.1 ± 1.37	6.56 ± 0.98	0.168 ± 0.021	0.440 ± 0.045	9.16 ± 0.72
Nitragina	85.0 ± 1.79	13.1 ± 1.41	6.57 ± 0.98	0.168 ± 0.022	0.439 ± 0.047	9.24 ± 0.67
Years						
2016	85.0 ^b^ ± 0.54	13.7 ^b^ ± 0.41	6.22 ^a^ ± 0.22	0.174 ^ab^ ± 0.007	0.450 ^b^ ± 0.016	9.59 ^b^ ± 0.27
2017	83.7 ^a^ ± 0.74	13.7 ^b^ ± 0.52	6.10 ^a^ ± 0.27	0.181 ^b^ ± 0.009	0.481 ^b^ ± 0.014	8.72 ^a^ ± 0.32
2018	86.2 ^b^ ± 2.31	12.2 ^a^ ± 2.13	7.30 ^b^ ± 1.44	0.154 ^a^ ± 0.032	0.413 ^a^ ± 0.070	8.46 ^a^ ± 0.47
2019	84.7 ^ab^ ± 1.29	13.4 ^ab^ ± 1.15	6.36 ^a^ ± 0.69	0.169 ^ab^ ± 0.017	0.429 ^a^ ± 0.032	10.00 ^c^ ± 0.28
Mean	84.9 ± 1.65	13.3 ± 1.38	6.50 ± 0.93	0.169 ± 0.021	0.443 ± 0.047	9.19 ± 0.72
Significances						
Cultivar	NS	NS	NS	NS	NS	*
Fertilisation	NS	NS	NS	NS	NS	NS
Inoculation	NS	NS	NS	NS	NS	NS
Years	**	*	**	**	**	***

* Mean values ± SD. DFA—index of desirable fatty acids; OFA—sum of hypercholesterolemic fatty acids; HH—hypocholesterolemic/hypercholesterolemic ratio; AI—index of atherogenicity; TI—index of thrombogenicity; CI—consumer index. Means in a column followed by different superscripts show significant differences (*p* < 0.05) according to the post-hoc Tukey test. Differences significant at: *** *p*  <  0.001; ** *p*  <  0.01; * *p*  <  0.05; NS not significant.

**Table 6:** The DFA, OFA, HH, AI, TI and CI indices of lipid quality in G. max seed; mean values for interaction cultivar × years.

Cultivar	Year	DFA	OFA	HH	AI	TI	CI
Aldana	2016	85.2 ± 0.46	13.4 ± 0.30	6.36 ± 0.17	0.170 ± 0.006	0.442 ± 0.013	9.39 ^c^ ± 0.20
	2017	84.1 ± 0.71	13.4 ± 0.30	6.30 ± 0.19	0.177 ± 0.008	0.473 ± 0.009	8.45 ^ab^ ± 0.13
	2018	86.9 ± 2.35	11.6 ± 2.08	7.74 ± 1.55	0.145 ± 0.031	0.383 ± 0.064	8.59 ^ab^ ± 0.58
	2019	84.5 ± 0.93	13.8 ± 0.64	6.13 ± 0.34	0.173 ± 0.011	0.437 ± 0.022	9.85 ^cd^ ± 0.29
Annushka	2016	84.7 ± 0.51	13.9 ± 0.33	6.08 ± 0.17	0.178 ± 0.006	0.459 ± 0.015	9.79 ^cd^ ± 0.18
	2017	83.3 ± 0.60	14.1 ± 0.40	5.91 ± 0.20	0.186 ± 0.007	0.488 ± 0.015	8.99 ^b^ ± 0.20
	2018	85.5 ± 2.19	12.8 ± 2.13	6.86 ± 1.25	0.162 ± 0.032	0.442± 0.067	8.33 ^a^ ± 0.33
	2019	85.0 ± 1.58	13.1 ± 1.44	6.59 ± 0.88	0.164 ± 0.022	0.420 ± 0.040	10.15 ^d^ ± 0.19
Significances							
Cultivar x Years		NS	NS	NS	NS	NS	*

* Mean values ± SD. DFA—index of desirable fatty acids; OFA—sum of hypercholesterolemic fatty acids; HH—hypocholesterolemic/hypercholesterolemic ratio; AI—index of atherogenicity; TI—index of thrombogenicity; CI—consumer index. Means in a column followed by different superscripts show significant differences (*p* < 0.05) according to the post-hoc Tukey test. Differences significant at: * *p*  <  0.05; NS not significant.

**Table 7:** The ODR, LDR and COX indices and the S/P, OL/(LA + ALA) ratio of lipid quality in *G. max* seeds; mean values for factors.

Factors	ODR	LDR	COX	S/P	OL/(LA + ALA)
Cultivars					
Aldana	75.4 ^a^ ± 3.01	14.6 ± 0.85	7.51^a^ ± 0.26	0.198 ^a^ ± 0.020	0.328 ^b^ ± 0.056
Annushka	77.5 ^b^ ± 2.38	14.6 ± 0.90	7.63 ^b^ ± 0.28	0.208 ^b^ ± 0.020	0.291 ^a^ ± 0.041
Fertilisation (kg∙ha^−1^ N)					
0	76.2 ± 3.33	14.6 ± 0.89	7.59 ± 0.24	0.198 ^a^ ± 0.026	0.314 ± 0.060
30	76.6 ± 2.80	14.6 ± 0.83	7.59 ± 0.27	0.204 ^ab^ ± 0.019	0.308 ± 0.050
60	76.6 ± 2.66	14.6 ± 0.92	7.53 ± 0.32	0.207 ^b^ ± 0.015	0.307 ± 0.047
Inoculation					
Without inoculation	76.4 ± 3.36	14.6 ± 0.91	7.59 ± 0.21	0.208 ^b^ ± 0.020	0.311 ± 0.061
HiStick^®^Soy	76.3 ± 2.94	14.6 ± 0.84	7.56 ± 0.33	0.198 ^a^ ± 0.023	0.312 ± 0.052
Nitragina	76.7 ± 2.47	14.6 ± 0.89	7.57 ± 0.29	0.203 ^ab^ ± 0.017	0.306 ± 0.043
Years					
2016	79.0 ^c^ ± 1.36	14.8 ^b^ ± 0.22	7.83 ^b^ ± 0.11	0.206 ^b^ ± 0.007	0.267 ^a^ ± 0.022
2017	76.5 ^b^ ± 0.75	14.0 ^a^ ± 0.42	7.39 ^a^ ± 0.06	0.214 ^c^ ± 0.009	0.308 ^b^ ± 0.013
2018	72.5 ^a^ ± 2.52	13.9 ^a^ ± 0.65	7.30 ^a^ ± 0.24	0.185 ^a^ ± 0.029	0.381 ^c^ ± 0.048
2019	77.9 ^c^ ± 1.06	15.8 ^c^ ± 0.33	7.77 ^b^ ± 0.15	0.207 ^bc^ ± 0.015	0.284 ^a^ ± 0.018
Mean	76.5 ± 2.91	14.6 ± 0.87	7.57 ± 0.28	0.203 ± 0.020	0.310 ± 0.052
Sifnificances					
Cultivar	***	NS	**	***	***
Fertilisation	NS	NS	NS	**	NS
Inoculation	NS	NS	NS	**	NS
Year	***	***	***	***	***

* Mean values ± SD. ODR—oleic desaturation ratio; LDR—linoleic desaturation ratio; COX—calculated oxidizability value; S/P—saturation fat index; OL/(LA+ALA)—18: 1/(18:2 + C18:3) acids ratio. Means in a column followed by different superscripts show significant differences (*p* < 0.05) according to the post-hoc Tukey test. Differences significant at: *** *p*  <  0.001; ** *p*  <  0.01; NS not significant.

**Table 8:** The ODR, LDR and COX indices and the S/P, OL/(LA+ALA) ratio of lipid quality in *G. max* seeds; mean values for interaction cultivar × years.

Cultivar	Year	ODR	LDR	COX	S/P	OL/(LA+ALA)
Aldana	2016	77.7 ^cd^ ± 0.45	14.7 ^bc^ ± 0.25	7.74 ± 0.06	0.201 ^bc^ ± 0.004	0.287 ^bc^ ± 0.007
	2017	76.0 ^bc^ ± 0.53	13.6 ^a^ ± 0.13	7.36 ± 0.07	0.206 ^bc^ ± 0.004	0.317 ^cd^ ± 0.009
	2018	70.7 ^a^ ± 1.98	14.4 ^b^ ± 0.54	7.26 ± 0.28	0.174 ^a^ ± 0.027	0.415 ^e^ ± 0.039
	2019	77.2 ^cd^ ± 1.04	15.7 ^d^ ± 0.42	7.69 ± 0.13	0.211 ^cd^ ± 0.010	0.296 ^bc^ ± 0.017
Annushka	2016	80.2 ^e^ ± 0.36	14.9 ^c^ ± 0.15	7.92 ± 0.06	0.211 ^cd^ ± 0.006	0.247 ^a^ ± 0.006
	2017	77.0 ^cd^ ± 0.55	14.4 ^b^ ± 0.14	7.41 ± 0.05	0.222 ^d^ ± 0.005	0.299 ^bc^ ± 0.009
	2018	74.3 ^b^ ± 1.60	13.4 ^a^ ± 0.29	7.35 ± 0.20	0.196 ^b^ ± 0.029	0.347 ^d^ ± 0.030
	2019	78.6 ^de^ ± 0.36	15.8 ^d^ ± 0.22	7.85 ± 0.12	0.203 ^bc^ ± 0.019	0.272 ^ab^ ± 0.006
Significances						
Cultivar x Years		*	***	NS	***	*

* Mean values ± SD. ODR—oleic desaturation ratio; LDR—linoleic desaturation ratio; COX—calculated oxidizability value; S/P—saturation fat index; OL/(LA+ALA)—18: 1/(18:2 + C18:3) acids ratio. Means in a column followed by different superscripts show significant differences (*p* < 0.05) according to the post-hoc Tukey test. Differences significant at: *** *p*  <  0.001; * *p*  <  0.05; NS not significant.

**Table 9:** The ODR, LDR and COX indices and the S/P, OL/(LA+ALA) ratio of lipid quality in *G. max* seeds; mean values for interaction cultivar × seed inoculation.

Cultivar	Inoculation	ODR	LDR	COX	S/P	OL/(LA+ALA)
Aldana	Without inoculation	75.2 ± 3.83	14.5 ± 0.99	7.55 ^ab^ ± 0.18	0.199 ^ab^ ± 0.022	0.334 ± 0.072
	HiStick^®^ Soy	75.3 ± 2.91	14.5 ± 0.68	7.54 ^ab^ ± 0.32	0.193 ^a^ ± 0.023	0.330 ± 0.053
	Nitragina	75.7 ± 2.36	14.7 ± 0.92	7.45 ^a^ ±0.28	0.203 ^a^ ± 0.015	0.322 ± 0.042
Annushka	Without inoculation	77.7 ± 2.34	14.7 ± 0.86	7.63 ^b^ ± 0.24	0.218 ^c^ ± 0.014	0.289 ± 0.040
	HiStick^®^ Soy	77.3 ± 2.70	14.6 ± 1.00	7.58 ^ab^ ± 0.36	0.203 ^ab^ ± 0.022	0.294 ± 0.047
	Nitragina	77.6 ± 2.30	14.5 ± 0.90	7.69 ^b^ ± 0.25	0.203 ^ab^ ± 0.019	0.290 ± 0.039
Sifnificances						
Cultivar x Inoculation		NS	NS	*	**	NS

* Mean values ± SD. ODR—oleic desaturation ratio; LDR—linoleic desaturation ratio; COX—calculated oxidizability value; S/P—saturation fat index; OL/(LA+ALA)—18: 1/(18:2 + C18:3) acids ratio. Means in a column followed by different superscripts show significant differences (*p* < 0.05) according to the post-hoc Tukey test. Differences significant at: ** *p*  <  0.01; * *p*  <  0.05; NS not significant.

## Data Availability

The data presented in this study are available in this article.

## Supplementary Materials

The following supporting information can be downloaded at: https://www.mdpi.com/article/10.3390/foods11050762/s1, Table S1: The content of Omega 3 and Omega 6 (g FA 100 g seeds−1) and also the ratio of Omega 6/Omega 3, MUFA/PUFA, PUFA/SFA, UFA/SFA and ALA/LA in G. max seeds; mean values for interaction nitrogen fertiliser application × years.; Table S2: The content of Omega 3 and Omega 6 (g FA 100 g seeds−1) as well as the ratio of Omega 6/Omega 3, MUFA/PUFA, PUFA/SFA, UFA/SFA and ALA/LA in G. max seeds; mean values for interaction inoculation × years; Table S3: The content of Omega 3 and Omega 6 (g FA 100 g seeds−1) as well as the ratio of Omega 6/Omega 3, MUFA/PUFA, PUFA/SFA, UFA/SFA and ALA/LA in G. max seeds; mean values for interaction cultivar × nitrogen fertiliser application; Table S4: The content of Omega 3 and Omega 6 (g FA 100 g seeds−1) as well as the ratio of Omega 6/Omega 3, MUFA/PUFA, PUFA/SFA, UFA/SFA and ALA/LA in G. max seeds; mean values for interaction cultivar x seed inoculation; Table S5: The content of Omega 3 and Omega 6 (g FA 100 g seeds−1) as well as the ratio of Omega 6/Omega 3, MUFA/PUFA, PUFA/SFA, UFA/SFA and ALA/LA in G. max seeds; mean values for interaction nitrogen fertiliser application × inoculation; Table S6: The DFA, OFA, HH, AI, TI and CI indices of lipid quality in G. max seed; mean values for interaction nitrogen fertiliser application × years; Table S7: The DFA, OFA, HH, AI, TI and CI indices of lipid quality in G. max seed; mean values for interaction inoculation × years; Table S8: The DFA, OFA, HH, AI, TI and CI indices of lipid quality in G. max seed; mean values for interaction cultivar × nitrogen fertiliser application; Table S9: The DFA, OFA, HH, AI, TI and CI indices of lipid quality in G. max seed; mean values for interaction cultivar x inoculation; Table S10: The DFA, OFA, HH, AI, TI and CI indices of lipid quality in G. max seed; mean values for interaction nitrogen fertiliser application × inoculation; Table S11: The ODR, LDR and COX indices and the S/P, OL/(LA+ALA) ratio of lipid quality in G. max seeds; mean values for interaction cultivar × nitrogen fertiliser application; S12: The ODR, LDR and COX indices and the S/P, OL/(LA+ALA) ratio of lipid quality in G. max seeds; mean values for interaction nitrogen fertiliser application × years; Table S13: The ODR, LDR and COX indices and the S/P, OL/(LA+ALA) ratio of lipid quality in G. max seeds; mean values for interaction inoculation × years; Table S14: The ODR, LDR and COX indices and the S/P, OL/(LA+ALA) ratio of lipid quality in G. max seeds; mean values for interaction nitrogen fertiliser application × inoculation.
