# Supplementary material for: Quality Evaluation Indices for Soybean Oil in Relation to Cultivar, Application of N Fertiliser and Seed Inoculation with Bradyrhizobium japonicum
Source: Foods. 2022 Mar 6;11(5):762. doi: 10.3390/foods11050762 (PMC8909349; doi:10.3390/foods11050762)
Supplement: Supplementary file 1 [file foods-11-00762-s001.zip › foods-1556852-supplementary.pdf]

foods MDPI

Article

Quality evaluation indices for soybean oil in relation to cultivar, application of N fertiliser and seed inoculation with *Bradyrhizobium japonicum*

Ewa Szpunar-Krok [1,]*, and Anna Wondołowska-Grabowska

Table S1. The content of Omega 3 and Omega 6 (g FA 100 g seeds^{-1}) as well as the ratio of Omega 6/Omega 3, MUFA/PUFA, PUFA/SFA, UFA/SFA and ALA/LA in *G. max* seeds; mean values for interaction nitrogen fertiliser application × years.

Fertilisation (kg·ha^{-1} N)	Years	Omega 3	Omega 6	Omega 6 / Omega 3	MUFA / PUFA	UFA / SFA	PUFA / SFA	ALA / LA
0	2016	9.58 ± 0.19	54.9 ± 0.61	5.74 ± 0.07	0.269 ± 0.023	4.55 ± 0.22	3.58 ± 0.13	0.173 ±0.002
	2017	8.61 ± 0.35	52.4 ± 0.63	6.10 ± 0.30	0.316 ± 0.013	4.11 ± 0.25	3.12 ± 0.16	0.160 ± 0.006
	2018	8.18 ± 0.63	51.2 ± 1.75	6.28 ± 0.32	0.405 ± 0.056	5.20 ± 0.99	3.69 ± 0.66	0.157 ± 0.009
	2019	9.94 ± 0.35	53.1 ± 0.90	5.35 ± 0.13	0.290 ± 0.017	4.40 ± 0.35	3.41 ± 0.28	0.185 ± 0.005
30	2016	9.44 ± 0.15	54.7 ± 0.52	5.79 ± 0.09	0.271 ± 0.018	4.41 ± 0.17	3.47 ± 0.09	0.171 ± 0.002
	2017	8.61 ± 0.29	52.3 ± 0.49	6.08 ± 0.21	0.317 ± 0.015	4.08 ± 0.13	3.10 ± 0.09	0.160 ± 0.006
	2018	8.54 ± 0.42	51.5 ± 1.65	6.04 ± 0.30	0.379 ± 0.046	4.92 ± 0.88	3.56 ± 0.57	0.163 ± 0.010
	2019	9.92 ± 0.29	53.0 ± 1.37	5.35 ± 0.15	0.289 ± 0.022	4.34 ± 0.45	3.37 ± 0.35	0.186 ± 0.005
60	2016	9.48 ± 0.43	54.7 ± 1.02	5.78 ± 0.21	0.277 ± 0.027	4.56 ± 0.07	3.57 ± 0.08	0.170 ± 0.004
	2017	8.62 ± 0.31	52.5 ± 0.47	6.09 ± 0.27	0.311 ± 0.012	4.05 ± 0.24	3.09 ± 0.15	0.160 ± 0.006
	2018	8.54 ± 0.30	52.2 ± 2.30	6.12 ± 0.31	0.374 ± 0.047	5.08 ± 0.45	3.70 ± 0.36	0.161 ± 0.009
	2019	10.05 ± 0.26	53.2 ± 0.93	5.30 ± 0.13	0.288 ± 0.017	4.42 ± 0.22	3.43 ± 0.19	0.187 ± 0.005
Inoculation × Years		NS	NS	NS	NS	NS	NS	NS

* Mean values ± SD. MUFA – monounsaturated fatty acids; PUFA– polyunsaturated fatty acids; UFA– unsaturated fatty acids; SFA– saturated fatty acids; ALA/LA – C18:3 n-3/C18:2 n-6 acid ratio. NS - not significant ($p < 0.05$).

Table S2. The content of Omega 3 and Omega 6 (g FA 100 g seeds^{-1}) as well as the ratio of Omega 6/Omega 3, MUFA/PUFA, PUFA/SFA, UFA/SFA and ALA/LA in *G. max* seeds; mean values for interaction inoculation × years.

Inoculation	Years	Omega 3	Omega 6	Omega 6/ Omega 3	MUFA / PUFA	UFA / SFA	PUFA / SFA	ALA / LA
Without inoculation	2016	9.51 ± 0.34	54.6 ± 0.43	5.75 ± 0.16	0.273 ± 0.025	4.47 ± 0.24	3.51 ±0.13	0.172 ± 0.003
	2017	8.58 ± 0.29	52.6 ± 0.32	6.14 ± 0.23	0.310 ± 0.013	4.07 ± 0.23	3.10 ± 0.15	0.161 ± 0.006
	2018	8.31 ± 0.53	51.0 ± 1.69	6.14 ± 0.27	0.400 ± 0.066	4.97 ± 0.84	3.54 ± 0.52	0.159 ± 0.008
	2019	10.06 ± 0.23	53.3 ± 0.74	5.30 ± 0.11	0.285 ± 0.015	4.40 ± 0.26	3.42 ± 0.20	0.188 ± 0.004
HiStick®Soy	2016	9.55 ± 0.17	54.8 ± 1.02	5.73 ± 0.06	0.271 ± 0.023	4.51 ± 0.12	3.55 ± 0.10	0.172± 0.001
	2017	8.57 ± 0.28	52.4 ± 0.35	6.12 ±0.23	0.315 ± 0.012	4.07 ± 0.19	3.10 ± 0.12	0.160 ± 0.005
	2018	8.35 ± 0.27	51.5 ± 2.39	6.17 ± 0.25	0.391 ± 0.045	5.06 ± 0.78	3.64 ± 0.56	0.160 ± 0.007
	2019	9.89 ± 0.41	53.1 ± 0.71	5.38 ± 0.18	0.294 ± 0.017	4.46 ± 0.29	3.44 ± 0.23	0.184 ± 0.006
Nitragina	2016	9.43 ± 0.33	54.9 ± 0.68	5.83 ± 0.14	0.273 ± 0.022	4.55 ± 0.16	3.57 ± 0.11	0.170 ± 0.005
	2017	8.70 ± 0.36	52.2 ± 0.73	6.01 ± 0.30	0.319 ± 0.015	4.10 ± 0.22	3.11 ± 0.15	0.160 ± 0.006
	2018	8.60 ± 0.59	52.4 ± 1.35	6.12 ± 0.44	0.367 ± 0.034	5.17 ± 0.81	3.78 ± 0.53	0.162 ± 0.012
	2019	9.96 ± 0.21	52.9 ± 1.56	5.31 ± 0.09	0.288 ± 0.022	4.31 ± 0.46	3.35 ± 0.38	0.186 ± 0.004
Inoculation × Years		NS	NS	NS	NS	NS	NS	NS

* Mean values ± SD. MUFA – monounsaturated fatty acids; PUFA– polyunsaturated fatty acids; UFA– unsaturated fatty acids; SFA– saturated fatty acids; ALA/LA – C18:3 n-3/C18:2 n-6 acid ratio. NS - not significant ($p < 0.05$).

Table S3. The content of Omega 3 and Omega 6 (g FA 100 g seeds^{-1}) as well as the ratio of Omega 6/Omega 3, MUFA/PUFA, PUFA/SFA, UFA/SFA and ALA/LA in *G. max* seeds; mean values for interaction cultivar × nitrogen fertiliser application.

Cultivar	Fertilisation (kg·ha^{-1} N)	Omega 3	Omega 6	Omega 6/Omega 3	MUFA/PUFA	UFA/SFA	PUFA / SFA	ALA / LA
Aldana	0	8.97 ± 0.83	52.7 ± 1.81	5.91 ± 0.42	0.340 ± 0.067	4.80 ± 0.71	3.58 ± 0.43	0.168 ± 0.013
	30	9.01 ± 0.60	52.2 ± 1.67	5.82 ± 0.34	0.333 ± 0.056	4.51 ± 0.69	3.38 ± 0.42	0.170 ± 0.011
	60	9.03 ± 0.67	52.5 ± 1.49	5.84 ± 0.41	0.331 ± 0.048	4.54 ± 0.42	3.41 ± 0.25	0.170 ± 0.013
Annushka	0	9.18 ± 0.83	53.1 ± 1.64	5.82 ± 0.44	0.301 ± 0.049	4.32 ± 0.52	3.32 ± 0.36	0.169 ± 0.013
	30	9.24 ± 0.70	53.5 ± 1.31	5.81 ± 0.37	0.295 ± 0.035	4.37 ± 0.43	3.37 ± 0.32	0.170 ± 0.012
	60	9.32 ± 0.75	53.8 ± 1.51	5.81 ± 0.42	0.294 ± 0.040	4.51 ± 0.52	3.49 ± 0.37	0.169 ± 0.013
Cultivar × Fertilisation		NS	NS	NS	NS	NS	NS	NS

* Mean values± SD. MUFA – monounsaturated fatty acids; PUFA– polyunsaturated fatty acids; UFA– unsaturated fatty acids; SFA– saturated fatty acids; ALA/LA – C18:3 n-3/C18:2 n-6 acid ratio. NS - not significant ($p < 0.05$).

Table S4. The content of Omega 3 and Omega 6 (g FA 100 g seeds^{-1}) as well as the ratio of Omega 6/Omega 3, MUFA/PUFA, PUFA/SFA, UFA/SFA and ALA/LA in *G. max* seeds; mean values for interaction cultivar × seed inoculation.

Cultivar	Inoculation	Omega 3	Omega 6	Omega 6/ Omega 3	MUFA / PUFA	UFA / SFA	PUFA / SFA	ALA / LA
Aldana	Without inoculation	8.94 ± 0.84	52.4 ± 1.82	5.90 ± 0.45	0.340 ± 0.072	4.65 ± 0.66	3.46 ± 0.38	0.169 ± 0.014
	HiStick® Soy	8.92 ± 0.62	52.3 ± 1.77	5.88 ±0.31	0.336 ± 0.053	4.52 ± 0.57	3.39 ± 0.38	0.168 ± 0.009
	Nitragina	9.14 ± 0.62	52.8 ± 1.33	5.79 ± 0.41	0.328 ± 0.043	4.69 ± 0.65	3.52 ± 0.40	0.171 ± 0.013
Annushka	Without inoculation	9.29 ± 0.74	53.3 ± 1.31	5.77 ± 0.35	0.294 ± 0.039	4.30 ± 0.36	3.32 ± 0.27	0.170 ± 0.012
	HiStick® Soy	9.25 ± 0.79	53.6 ± 1.53	5.83 ± 0.45	0.300 ± 0.047	4.53 ± 0.53	3.48 ± 0.35	0.170 ± 0.014
	Nitragina	9.20 ± 0.76	53.5 ± 1.69	5.84 ± 0.43	0.296 ± 0.038	4.38 ± 0.55	3.38 ± 0.42	0.169 ± 0.012
Cultivar x Inoculation		NS	NS	NS	NS	NS	NS	NS

* Mean values ± SD. MUFA – monounsaturated fatty acids; PUFA– polyunsaturated fatty acids; UFA– unsaturated fatty acids; SFA– saturated fatty acids; ALA/LA – C18:3 n-3/C18:2 n-6 acid ratio. NS - not significant ($p < 0.05$).

Table S5. The content of Omega 3 and Omega 6 (g FA 100 g seeds^{-1}) as well as the ratio of Omega 6/Omega 3, MUFA/PUFA, PUFA/SFA, UFA/SFA and ALA/LA in *G. max* seeds; mean values for interaction nitrogen fertiliser application × inoculation.

Fertilisation (kg·ha^{-1} N)	Inoculation	Omega 3	Omega 6	Omega 6/ Omega 3	MUFA / PUFA	UFA / SFA	PUFA / SFA	ALA / LA
0	Without inoculation	8.98 ± 0.93	52.7 ± 2.14	5.90 ± 0.46	0.322 ± 0.074	4.38 ± 0.31	3.32 ± 0.22	0.168 ± 0.014
	HiStick®Soy	9.12 ± 0.77	53.1 ± 1.44	5.85 ± 0.40	0.326 ± 0.061	4.78 ± 0.76	3.60 ± 0.44	0.170 ± 0.012
	Nitra-gina	9.13 ± 0.85	53.0 ± 1.65	5.85 ± 0.47	0.313 ± 0.054	4.53 ± 0.80	3.44 ± 0.52	0.169 ± 0.013
30	Without inoculation	9.10 ± 0.68	53.0 ± 1.39	5.84 ± 0.34	0.309 ± 0.058	4.40 ± 0.75	3.35 ± 0.44	0.170 ± 0.011
	HiStick®Soy	9.01 ± 0.76	52.7 ± 1.85	5.88 ± 0.41	0.316 ± 0.049	4.31 ± 0.25	3.28 ± 0.23	0.169 ± 0.013
	Nitragina	9.27 ± 0.57	53.0 ± 1.73	5.73 ± 0.33	0.316 ± 0.046	4.60 ± 0.62	3.49 ± 0.41	0.171 ± 0.012
60	Without inoculation	9.26 ± 0.83	53.0 ± 1.44	5.75 ± 0.43	0.319 ± 0.058	4.64 ± 0.54	3.51 ± 0.30	0.171 ± 0.014
	HiStick®Soy	9.13 ± 0.71	53.1 ± 2.13	5.83 ± 0.37	0.312 ± 0.052	4.48 ± 0.43	3.42 ± 0.35	0.169 ± 0.011
	Nitragina	9.12 ± 0.68	53.4 ± 1.38	5.88 ± 0.46	0.305 ± 0.031	4.46 ± 0.44	3.42 ± 0.31	0.169 ± 0.014
Fertilisation × Inoculation		NS	NS	NS	NS	NS	NS	NS

* Mean values ± SD. MUFA – monounsaturated fatty acids; PUFA– polyunsaturated fatty acids; UFA– unsaturated fatty acids; SFA– saturated fatty acids; ALA/LA – C18:3 n-3/C18:2 n-6 acid ratio. NS - not significant ($p < 0.05$).

Table S6. The DFA, OFA, HH, AI, TI and CI indices of lipid quality in G. max seed; mean values for interaction nitrogen fertiliser application × years.

Fertilisation (kg·ha^{-1} N)	Years	DFA	OFA	HH	IA	IT	CI
0	2016	85,1 ± 0,66	13,6 ± 0,42	6,28 ± 0,24	0,173 ± 0,009	0,446 ± 0,017	9,66 ± 0,20
	2017	83,8 ± 0,88	13,8 ± 0,70	6,07 ± 0,36	0,182 ± 0,011	0,483 ± 0,020	8,73 ± 0,40
	2018	86,5 ± 2,88	11,6 ± 2,48	7,77 ± 1,87	0,148 ± 0,037	0,403 ± 0,085	8,25 ± 0,65
	2019	84,8 ± 1,37	13,3 ± 1,09	6,41 ± 0,67	0,167 ± 0,017	0,427 ± 0,035	9,98 ± 0,34
30	2016	84,7 ± 0,56	13,9 ± 0,52	6,10 ± 0,27	0,178 ± 0,008	0,458 ± 0,017	9,53 ± 0,18
	2017	83,7 ± 0,58	13,7 ± 0,19	6,13 ± 0,10	0,182 ± 0,005	0,478 ± 0,009	8,72 ± 0,27
	2018	85,7 ± 2,71	12,8 ± 2,69	6,98 ± 1,60	0,162 ± 0,041	0,426 ± 0,092	8,56 ± 0,42
	2019	84,5 ± 1,71	13,6 ± 1,53	6,28 ± 0,94	0,172 ± 0,024	0,436 ± 0,039	9,95 ± 0,29
60	2016	85,2 ± 0,16	13,6 ± 0,23	6,27 ± 0,11	0,172 ± 0,004	0,446 ± 0,014	9,57 ± 0,42
	2017	83,5 ± 0,86	13,7 ± 0,60	6,11 ± 0,34	0,180 ± 0,010	0,480 ± 0,012	8,71 ± 0,34
	2018	86,4 ± 1,43	12,2 ± 1,12	7,15 ± 0,78	0,152 ± 0,019	0,409 ± 0,031	8,56 ± 0,30

	2019	84,9 ± 0,88	13,4 ± 0,95	6,38 ± 0,52	0,167 ± 0,011	0,423 ± 0,025	10,07 ± 0,25
Inoculation × Years		NS	NS	NS	NS	NS	NS

* Mean values ± SD. DFA - index of desirable fatty acids; OFA – sum of hypercholesterolemic fatty acids; HH - hypocholesterolemic/hypercholesterolemic ratio; AI - index of atherogenicity; TI - index of thrombogenicity; CI - consumer index. NS - not significant ($p < 0.05$).

Table S7. The DFA, OFA, HH, AI, TI and CI indices of lipid quality in *G. max* seed; mean values for interaction inoculation × years.

Inoculation	Years	DFA	OFA	HH	IA	IT	CI
Without inoculation	2016	84,9 ± 0,77	13,8 ± 0,57	6,15 ± 0,31	0,176 ± 0,010	0,454 ± 0,019	9,60 ± 0,35
	2017	83,6 ± 0,94	13,9 ± 0,65	6,05 ± 0,33	0,182 ± 0,011	0,486 ± 0,019	8,67 ± 0,29
	2018	85,9 ± 2,53	12,8 ± 2,55	6,98 ± 1,52	0,163 ± 0,039	0,429 ± 0,086	8,36 ± 0,51
	2019	84,8 ± 1,03	13,7 ± 0,81	6,23 ± 0,45	0,171 ± 0,013	0,432 ± 0,027	10,08 ± 0,22
HiStick®Soy	2016	85,0 ± 0,36	13,7 ± 0,29	6,22 ± 0,15	0,174 ± 0,005	0,447 ± 0,015	9,66 ± 0,16
	2017	83,7 ± 0,63	13,8 ± 0,43	6,08 ± 0,23	0,182 ± 0,007	0,484 ± 0,010	8,67 ± 0,30
	2018	86,3 ± 2,37	12,1 ± 2,18	7,40 ± 1,61	0,151 ± 0,032	0,409 ± 0,062	8,38 ± 0,27
	2019	85,0 ± 1,12	13,1 ± 1,16	6,56 ± 0,69	0,163 ± 0,017	0,421 ± 0,033	9,92 ± 0,42
Nitragina	2016	85,1 ± 0,46	13,6 ± 0,36	6,28 ± 0,20	0,172 ± 0,006	0,450 ± 0,016	9,51 ± 0,30
	2017	83,8 ± 0,76	13,6 ± 0,50	6,19 ± 0,28	0,180 ± 0,010	0,473 ± 0,009	8,82 ± 0,39
	2018	86,5 ± 2,44	11,8 ± 1,91	7,52 ± 1,41	0,147 ± 0,028	0,400 ± 0,071	8,64 ± 0,61
	2019	84,4 ± 1,75	13,6 ± 1,50	6,29 ± 0,94	0,172 ± 0,023	0,434 ± 0,040	9,99 ± 0,19
Inoculation × Years		NS	NS	NS	NS	NS	NS

* Mean values ± SD. DFA - index of desirable fatty acids; OFA – sum of hypercholesterolemic fatty acids; HH - hypocholesterolemic/hypercholesterolemic ratio; AI - index of atherogenicity; TI - index of thrombogenicity; CI - consumer index. NS - not significant ($p < 0.05$).

Table S8. The DFA, OFA, HH, AI, TI and CI indices of lipid quality in *G. max* seed; mean values for interaction cultivar × nitrogen fertiliser application.

Cultivar	Fertilisation ($kg \cdot ha^{-1}$ N)	DFA	OFA	HH	IA	IT	CI
Aldana	0	85,8 ± 1,78	12,6 ± 1,59	6,94 ± 1,28	0,160 ± 0,023	0,424 ± 0,053	9,04 ± 0,80
	30	84,7 ± 1,96	13,3 ± 1,53	6,49 ± 1,05	0,171 ± 0,024	0,440 ± 0,053	9,08 ± 0,59
	60	85,0 ± 1,18	13,2 ± 0,90	6,46 ± 0,61	0,168 ± 0,014	0,438 ± 0,034	9,09 ± 0,66
Annushka	0	84,3 ± 1,74	13,5 ± 1,50	6,33 ± 1,01	0,175 ± 0,023	0,456 ± 0,052	9,27 ± 0,84
	30	84,6 ± 1,47	13,7 ± 1,56	6,26 ± 0,87	0,175 ± 0,024	0,459 ± 0,051	9,30 ± 0,71

	60	85,0 ± 1,59	13,2 ± 1,07	6,49 ± 0,65	0,167 ± 0,018	0,442 ± 0,035	9,38 ± 0,75
Cultivar × Fertilisation		NS	NS	NS	NS	NS	NS

* Mean values ± SD. DFA - index of desirable fatty acids; OFA – sum of hypercholesterolemic fatty acids; HH - hypocholesterolemic/hypercholesterolemic ratio; AI - index of atherogenicity; TI - index of thrombogenicity; CI - consumer index. NS - not significant ($p < 0.05$).

Table S9. The DFA, OFA, HH, AI, TI and CI indices of lipid quality in *G. max* seed; mean values for interaction cultivar × inoculation.

Cultivar	Inoculation	DFA	OFA	HH	IA	IT	CI
Aldana	Without inoculation	85,3 ± 1,83	13,0 ± 1,39	6,64 ± 0,99	0,165 ± 0,021	0,435 ± 0,050	9,00 ± 0,82
	HiStick® Soy	84,8 ± 1,62	13,2 ± 1,31	6,51 ± 1,01	0,169 ± 0,020	0,440 ± 0,043	9,00 ± 0,62
	Nitragina	85,4 ± 1,71	12,9 ± 1,50	6,74 ± 1,11	0,164 ± 0,022	0,427 ± 0,050	9,22 ± 0,58
Annushka	Without inoculation	84,3 ± 1,30	14,0 ± 1,23	6,06 ± 0,60	0,181 ± 0,020	0,466 ± 0,047	9,36 ± 0,74
	HiStick® Soy	85,1 ± 1,60	13,1 ± 1,48	6,62 ± 0,99	0,166 ± 0,023	0,440 ± 0,048	9,32 ± 0,80
	Nitragina	84,5 ± 1,83	13,4 ± 1,33	6,40 ± 0,85	0,171 ± 0,021	0,451 ± 0,043	9,26 ± 0,78
Cultivar x Inoculation		NS	NS	NS	NS	NS	NS

* Mean values ± SD. DFA - index of desirable fatty acids; OFA – sum of hypercholesterolemic fatty acids; HH - hypocholesterolemic/hypercholesterolemic ratio; AI - index of atherogenicity; TI - index of thrombogenicity; CI - consumer index. NS - not significant ($p < 0.05$).

Table S10. The DFA, OFA, HH, AI, TI and CI indices of lipid quality in *G. max* seed; mean values for interaction nitrogen fertiliser application × inoculation.

Fertilisation ($kg \cdot ha^{-1}$ N)	Inoculation	DFA	OFA	HH	AI	TI	CI
0	Without inoculation	84,6 ± 1,01	13,8 ± 0,68	6,17 ± 0,36	0,178 ± 0,012	0,460 ± 0,031	9,06 ± 0,91
	HiStick®Soy	85,8 ± 2,05	12,3 ± 1,95	7,16 ± 1,45	0,155 ± 0,028	0,418 ± 0,063	9,19 ± 0,77
	Nitragina	84,8 ± 2,30	13,2 ± 1,69	6,58 ± 1,30	0,169 ± 0,025	0,441 ± 0,059	9,21 ± 0,86
30	Without inoculation	84,4 ± 2,17	13,9 ± 1,95	6,22 ± 1,21	0,178 ± 0,030	0,463 ± 0,066	9,17 ± 0,68
	HiStick®Soy	84,3 ± 0,88	13,9 ± 0,57	6,09 ± 0,30	0,178 ± 0,010	0,462 ± 0,020	9,08 ± 0,77
	Nitragina	85,2 ± 1,84	12,7 ± 1,59	6,81 ± 1,03	0,164 ± 0,026	0,425 ± 0,054	9,33 ± 0,54
60	Without inoculation	85,4 ± 1,55	12,9 ± 1,19	6,67 ± 0,79	0,162 ± 0,019	0,428 ± 0,044	9,30 ± 0,83

	HiStick®Soy	84,8 ± 1,38	13,2 ± 0,81	6,45 ± 0,51	0,170 ± 0,015	0,440 ± 0,033	9,21 ± 0,71
	Nitragina	84,8 ± 1,28	13,5 ± 0,90	6,32 ± 0,54	0,170 ± 0,013	0,451 ± 0,024	9,18 ± 0,66
Fertilisation × Inoculation		NS	NS	NS	NS	NS	NS

* Mean values ± SD. DFA - index of desirable fatty acids; OFA – sum of hypercholesterolemic fatty acids; HH - hypocholesterolemic/hypercholesterolemic ratio; AI - index of atherogenicity; TI - index of thrombogenicity; CI - consumer index. NS - not significant ($p < 0.05$).

Table S11. The ODR, LDR, and COX indices and the S/P, OL/(LA+ALA) ratio of lipid quality in *G. max* seeds; mean values for interaction cultivar × nitrogen fertiliser application.

Cultivar	Fertilisation (kg·ha^{-1} N)	ODR	LDR	COX	S/P	OL/(LA+ALA)
Aldana	0	75,1 ± 3,54	14,5 ± 0,90	7,53 ± 0,19	0,196 ± 0,028	0,334 ± 0,066
	30	75,5 ± 3,08	14,6 ± 0,79	7,55 ± 0,24	0,198 ± 0,018	0,327 ± 0,056
	60	75,6 ± 2,60	14,6 ± 0,93	7,46 ± 0,34	0,201 ± 0,014	0,325 ± 0,048
Annushka	0	77,3 ± 2,83	14,6 ± 0,92	7,66 ± 0,27	0,201 ± 0,025	0,295 ± 0,049
	30	77,6 ± 2,11	14,6 ± 0,90	7,64 ± 0,30	0,211 ± 0,018	0,289 ± 0,035
	60	77,7 ± 2,36	14,6 ± 0,95	7,60 ± 0,30	0,213 ± 0,014	0,289 ± 0,040
Cultivar × Fertilisation		NS	NS	NS	NS	NS

* Mean values ± SD. ODR - oleic desaturation ratio; LDR - linoleic desaturation ratio; COX - calculated oxidizability value; S/P - saturation fat index; OL/(LA+ALA) - 18: 1/(18:2 + C18:3) acids ratio. NS - not significant ($p < 0.05$).

Table S12. The ODR, LDR, and COX indices and the S/P, OL/(LA+ALA) ratio of lipid quality in *G. max* seeds; mean values for interaction nitrogen fertiliser application × years.

Fertilisation (kg·ha^{-1} N)	Years	ODR	LDR	COX	OL/(LA+ALA)
0	2016	79.1 ± 1.45	14.9 ± 0.15	7.84 ± 0.08	0.264 ± 0.023
	2017	76.4 ± 0.79	14.0 ± 0.48	7.37 ± 0.09	0.309 ± 0.013
	2018	71.6 ± 2.86	13.7 ± 0.63	7.41 ± 0.09	0.399 ± 0.056
	2019	77.8 ± 1.00	15.7 ± 0.32	7.74 ± 0.20	0.285 ± 0.017
30	2016	79.1 ± 1.15	14.7 ± 0.21	7.82 ± 0.14	0.265 ± 0.018
	2017	76.4 ± 0.84	14.0 ± 0.39	7.41 ± 0.05	0.310 ± 0.014
	2018	72.9 ± 2.41	14.1 ± 0.69	7.31 ± 0.21	0.374 ± 0.046
	2019	77.9 ± 1.34	15.7 ± 0.35	7.83 ± 0.13	0.284 ± 0.022

60	2016	78.7 ± 1.65	14.7 ± 0.29	7.84 ± 0.12	0.271 ± 0.027
	2017	76.7 ± 0.70	13.9 ± 0.47	7.38 ± 0.05	0.304 ± 0.012
	2018	73.1 ± 2.46	13.9 ± 0.69	7.18 ± 0.34	0.369 ± 0.046
	2019	78.0 ± 1.02	15.9 ± 0.35	7.73 ± 0.07	0.282 ± 0.017
Inoculation × Years		NS	NS	NS	NS

* Mean values ± SD. ODR - oleic desaturation ratio; LDR - linoleic desaturation ratio; COX - calculated oxidizability value; OL/(LA+ALA) - 18: 1/(18:2 + C18:3) acids ratio. NS - not significant ($p < 0.05$).

Table S13. The ODR, LDR, and COX indices and the S/P, OL/(LA+ALA) ratio of lipid quality in *G. max* seeds; mean values for interaction inoculation × years.

Inoculation	Years	ODR	LDR	COX	OL/(LA+ALA)
Without inoculation	2016	78.9 ± 1.56	14.8 ± 0.22	7.79 ± 0.10	0.267 ± 0.025
	2017	76.7 ± 0.77	14.0 ± 0.43	7.42 ± 0.05	0.304 ± 0.013
	2018	71.8 ± 3.38	13.8 ± 0.54	7.38 ± 0.13	0.395 ± 0.066
	2019	78.2 ± 0.93	15.8 ± 0.27	7.76 ± 0.09	0.280 ± 0.015
HiStick®Soy	2016	79.0 ± 1.43	14.8 ± 0.12	7.90 ± 0.10	0.266 ± 0.023
	2017	76.5 ± 0.66	13.9 ± 0.44	7.38 ± 0.04	0.308 ± 0.011
	2018	72.2 ± 2.34	13.9 ± 0.55	7.19 ± 0.28	0.386 ± 0.044
	2019	77.6 ± 1.06	15.6 ± 0.45	7.77 ± 0.16	0.289 ± 0.018
Nitragina	2016	78.9 ± 1.36	14.6 ± 0.28	7.82 ± 0.11	0.267 ± 0.022
	2017	76.3 ± 0.88	14.0 ± 0.47	7.36 ± 0.08	0.311 ± 0.015
	2018	73.5 ± 1.75	14.0 ± 0.89	7.34 ± 0.29	0.362 ± 0.033
	2019	78.0 ± 1.29	15.8 ± 0.25	7.77 ± 0.19	0.283 ± 0.021
Inoculation × Years		NS	NS	NS	NS

* Mean values ± SD. ODR - oleic desaturation ratio; LDR - linoleic desaturation ratio; COX - calculated oxidizability value; OL/(LA+ALA) - 18: 1/(18:2 + C18:3) acids ratio. NS - not significant ($p < 0.05$).

Table S14. The ODR, LDR, and COX indices and the S/P, OL/(LA+ALA) ratio of lipid quality in *G. max* seeds; mean values for interaction nitrogen fertiliser application × inoculation.

Fertilisation ($kg \cdot ha^{-1}$ N)	Inoculation	ODR	LDR	COX	OL/(LA+ALA)
0	Without inoculation	76.2 ± 3.91	14.5 ± 0.98	7.59 ± 0.22	0.316 ± 0.073
	HiStick®Soy	75.9 ± 3.43	14.6 ± 0.87	7.60 ± 0.27	0.320 ± 0.061

	Nitragina	76.6 ± 3.02	14.6 ± 0.94	7.59 ± 0.26	0.308 ± 0.053
30	Without inoculation	76.8 ± 3.29	14.6 ± 0.80	7.58 ± 0.23	0.303 ± 0.059
	HiStick®Soy	76.4 ± 2.75	14.5 ± 0.96	7.64 ± 0.30	0.310 ± 0.049
	Nitragina	76.4 ± 2.69	14.7 ± 0.82	7.56 ± 0.31	0.311 ± 0.047
60	Without inoculation	76.2 ± 3.25	14.7 ± 1.04	7.59 ± 0.21	0.315 ± 0.058
	HiStick®Soy	76.6 ± 2.96	14.6 ± 0.81	7.44 ± 0.42	0.307 ± 0.053
	Nitragina	77.0 ± 1.86	14.5 ± 1.01	7.56 ± 0.32	0.299 ± 0.031
Fertilisation × Inoculation		NS	NS	NS	NS

* Mean values ± SD. ODR - oleic desaturation ratio; LDR - linoleic desaturation ratio; COX - calculated oxidizability value; OL/(LA+ALA) - 18: 1/(18:2 + C18:3) acids ratio. NS - not significant ($p < 0.05$).
